# Time Reverse Modeling of Damage Detection in Underwater Concrete Beams Using Piezoelectric Intelligent Modules

**DOI:** 10.3390/s20247318

**Published:** 2020-12-19

**Authors:** Jiachen Liang, Bo Chen, Chenfei Shao, Jianming Li, Bangbin Wu

**Affiliations:** College of Water Conservancy and Hydropower Engineering, Hohai University, Nanjing 210098, China; jc.liang.hhu@gmail.com (J.L.); shao_chen_fei@126.com (C.S.); lijianming@hhu.edu.cn (J.L.); wubangbin1986@126.com (B.W.)

**Keywords:** time reversal, intelligent modules, concrete, underwater crack, health monitoring, wave propagation

## Abstract

Underwater cracks in concrete structures are often difficult to detect due to their complexity of the service environment. With numerical and experimental analysis of concrete beams immersed in water, an active monitoring system, based on a cement-based piezoelectric intelligent module array (CPIMA), was developed to locate and quantify the underwater cracks. Time reversal (TR) of the stress wave field is accomplished to focus on the crack area through the concrete beam specimen by the system. First, a piezoelectric actuator is applied to emit the initial propagating wave, which can be reflected, attenuated, and diffracted by the crack, transmitted through water filled in the crack, as well as diffracted by the coarse aggregates. To extract the damage waveforms associated with the crack and analyze the robust time-reversal invariance under the high-order multiple scattering effect, a pair of homogeneous and heterogeneous forward finite element (FE) models is established. Then, the damage waveforms are time-reversed and re-propagated in the inverse numerical model, where an optimal refocusing is achieved on the crack that behaves as an acoustic source. Finally, the damage area is obtained in the form of the stacked energy distribution of each time step. The focus results are represented by cloud images and compared with root-mean-square deviation (RMSD) values. Numerical simulation and experiments show that this method can identify and quantify underwater cracks effectively.

## 1. Introduction

As a porous solid colloid, concrete usually suffers from micro cracks resulting from pore water, pore air, and chemical admixtures in the early stages of construction. For underwater concrete structures, such as dams, pipelines, and bridge piers, these micro cracks may propagate to macro cracks under cyclic environmental loads, creep, and shrinkage [1,2]. To evaluate the damage degree of concrete structures and arrange remedial measures, it is often necessary to locate and quantify the depth of these cracks. In order to detect underwater cracks, the traditional optical image detection techniques have been used for decades. However, due to the loss of information (scattering, absorption, and convolution effects) in underwater conditions, the quality of underwater images is usually poor and the crack characteristics are difficult to be extracted. Other technologies, such as electrical impedance tomography [3], ground-penetrating radar testing [4], and vibration-based technique [5,6], are time, energy, and financially consuming, and they are easily affected by the noise caused by the complex underwater service environment and the heterogeneity of concrete.

In recent years, the ultrasonic pulse-based structural health monitoring (SHM) method has been widely used to detect the depth of underwater concrete cracks owing to the strong penetration ability and simplicity of operation [7,8]. However, the traditional crack detection method usually uses only a specific component of the crack-related information (energy attenuation, travel time of diffracted wave, or the scattered wave field). Due to the interference between each component, it is difficult to extract a certain component from noises, especially for complex situations, such as underwater problems. To the author’s best knowledge, there are few studies that make use of information from various wave components comprehensively. The time of flight (TOF)-based technique is probably the most commonly used approach in practice [9,10]. The TOF approach estimates the crack depth by measuring the travel time of the p-wave, which is generated by a pulse-velocity meter or an impact event on the concrete surface to be diffracted at the crack tip and captured by a receiver mounted on the surface [11]. Only when the wave paths are correctly assumed and a high sharpness definition of the crack tip as a diffractor of the elastic wave is guaranteed can it provide reasonable crack depth estimation [12]. When the crack is filled with water, however, waves can transmit through the media of water. The transmitted waves through water bypass the crack tip to the receiver, creating shorter wave paths than the assumed ones. Meanwhile, the sharpness of the stress wave diffractometer at the crack tip is limited since the diffracted wave from the crack tip can be interfered with or even submerged by the transmitted wave through water. Therefore, there are defects in the TOF-based technique when applying it to underwater cracks. For example, the ultrasonic inspection instrument, which is based on the TOF approach and recommended by the Chinese industry code for surface concrete crack depth detection, is prohibited from use in underwater cracks by the code [13,14]. Therefore, it is necessary to develop a method to remove the negative effect of the transmitted waves through water. Meanwhile, the above detection techniques are often difficult to be applied underwater, which requires a reliable and convenient monitoring system to stimulate and receive elastic waves.

With the advantage of low cost, low power consumption, fast response, and wide frequency response range, cement-based piezoelectric modules have arisen wide concern in SHM [15,16]. Compared to other transducers, it has high durability and good compatibility with concrete by encapsulating piezoelectric cores with cement cover [17,18,19]. There are two main SHM methods based on piezoelectric sensors: The impedance method and wave-based method. The impedance method utilizes the relationship between the opening status of cracks and the mechanical impedance of the test specimen. This method is more suitable for minor cracks and local damage monitoring. The wave-based method, due to its high sensitivity and long detection distance, has a wide range of applications for crack diagnosis of various underwater concrete structures.

Researchers have conducted a lot of studies on the electrical and mechanical properties of the piezoelectric module and the propagation characteristics of stress waves in concrete, laying a foundation for the use of the wave-based method for the piezoelectric module in concrete. Song et al. [20] first developed an embedded piezoelectric intelligent aggregate by encapsulating the piezoelectric transducer in marble, and studied its property of waterproof and electromagnetic shielding. The piezoelectric aggregates were then applied to detect the damage of reinforced concrete bridge girders [21]. The sensitivity of piezoelectric modules to different acoustic parameters, such as frequency, wave velocity, and phase change, was analyzed by Su et al. [22]. Zou [23] proposed a piezoceramic smart aggregate-based approach to investigate the relationship between the travel time (velocity) of P-waves and the water seepage depth in concrete beams. Based on the RMSD damage index, Feng [24] investigated the leakage of a concrete pipe based on the active monitoring method and piezoelectric sensors. The results show that the water filling in the crack functions as a medium for the propagating wave. Baharom [25] analyzed the effect of water pressure on the amplitude of the received signal of the Rayleigh wave captured by a gel-coupled piezoceramic sensor (GCP) and detected the underwater concrete cracks of different depths at different frequency bands using RMSD. Numerical simulations are of great importance for a better understanding of the waveforms associated with cracks, as well as their propagation through homogeneous or heterogeneous media. Nestorović et al. [26,27] established both the piezoelectric module and wave propagating numerical models to analyze the relationship between RMSD and different cracks with different sizes, positions, and directions. The results of both the simulation and experiment show the sensitivity of the wave energy attenuation method to the presence of concrete cracks. Unlike the scattered waves, however, the attenuated waves have seldom been used for crack imaging in previous studies since they it is hard to capture them directly.

To image underwater cracks, the time-reversal method (TRM) was herein introduced [28]. TRM is used to converge waves synchrony at the source (and possibly reflected or scattered by the target of interest) [29], which is widely applied in underwater communication, medical imaging, and nondestructive testing [30,31,32]. The beauty of this method is that it can retrace complex multipaths of damage signals through a high-order scattering medium, which effectively improves the signal-to-noise ratio of underwater concrete problems [29]. Prada C. et al. [33] proposed a decomposition method of the time reversal operator to achieve accurate energy focusing on the position of multiple point targets. Based on this, Ulrich T. J. et al. applied the time-inelastic nonlinear diagnosis (TREND) technique to locate damages at different depths in a concrete specimen [34,35]. Kocur G. K. [36] applied TRM and signal-based acoustic emission (AE) technology to locate concrete cracks. This method can use the waveform of received signals to locate the AE activity from cracks, and neglect the pre-selection process. Although TRM has been widely used to identify damage in concrete structures, it has rarely been reported to locate underwater concrete cracks. The accuracy of traditional diffracted wave-based TRM can suffer from being influenced by the transmitted wave through water in the crack. First, the transmitted wave through water creates direct wave paths from the actuator to the sensors, which can be retraced by backpropagating waves to focus on the actuator, rather than the crack. Second, as a wave-based method, the accuracy of TRM depends on the sharpness and strength of the wave source, which can be weakened by the interference of the transmitted wave through water. To overcome this problem, the damage-related waves (including reflected, attenuated, and diffracted waves) are proposed to make the crack a stronger signal source than the crack tip as a diffractor. The multiple paths of different wave components can be retraced by TRM; in this way, a constructive interference can be achieved on the wave source due to time-reversal invariance and the reciprocity theorem. In addition, the proposed damage-related wave considers the transmitted waves through water as a part of the attenuated wave component, in which way the negative effect of water in the crack is reduced.

In this paper, a TRM-based imaging technique is developed to detect underwater concrete cracks with the help of a piezoelectric-based monitoring system. The application of TRM is improved by using the damage-related waves and the crack information is better used. The feasibility and robustness of the proposed method is studied by the forward and inverse heterogeneous FE simulation, respectively. In the experiment, a group of concrete specimens with artificial cracks were immersed in water and tested by the CPIMA-based active monitoring system. Finally, the imaging results are discussed and compared with the energy-based RMSD results.

## 2. Piezoelectric Intelligent Module and Detecting Approach for Cracks

### 2.1. Piezoelectric Intelligent Module

**Figure 1:**
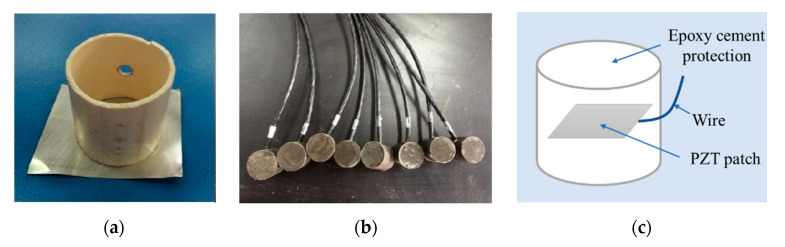
Fabrication of piezoelectric intelligent modules: (**a**) A homemade mold; (**b**) piezoelectric intelligent modules; (**c**) schematic of the module.

Piezoelectric intelligent modules are the basic monitoring components in this method. Since the piezoelectric patches are composed of a thin brittle material, they are encapsulated in cement as intelligent modules to enhance the durability and the compatibility with concrete [37]. In this paper, the intelligent module is made of an epoxy cement protection of 30 mm (diameter) × 30 mm (height), with a circular lead zirconate titanate (PZT) patch of a diameter of 20 mm (diameter) × 1 mm (thickness) inside (see Figure 1c). The production process is as follows: first, the PZT patch was placed in a self-made mold (see Figure 1a). Then, the epoxy cement was stirred evenly and poured into the mold. After, the mold was continuously vibrated and vacuumized to reduce the porosity of the cement. Finally, the obtained sample was placed in the standard curing chamber for 7 days, with the control temperature at (20 ± 1) °C and the relative humidity ≥90%.

### 2.2. Wave-Based SHM Method

**Figure 2:**
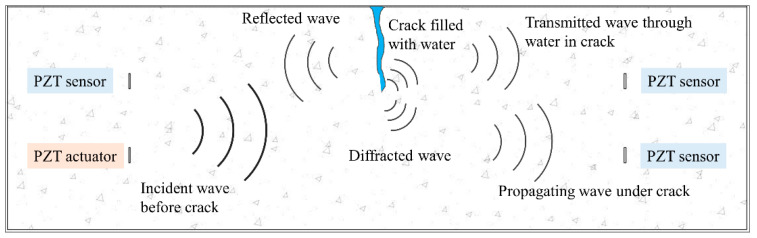
Schematic of stress waves propagating through a cracked concrete beam.

Because of the direct and inverse piezoelectric effect, the piezoelectric intelligent modules can be used as sensors, which can record real-time stress waves as voltage signals, and can also function as actuators to stimulate modulated signals. For the self-made piezoelectric intelligent module, the stimulated signal would propagate in the form of both P-waves and S-waves through solid materials. The P-wave can travel across a long distance along the polarization direction. Since the good orientation of the P-wave can help to form more effective propagating paths between CPIMA, the P-wave is herein simulated. If there is a crack in this propagation path, the traveling wave will be blocked, reflected, and diffracted. According to Huygens’ principle, all these influences behave as secondary wave fronts, which originate from the crack, see Figure 2.

Concrete is a typical heterogeneous material since it is composed of aggregates, mortar, and cement matrix. When waves are propagating through such heterogeneous mediums, noises are generated due to refraction, diffraction, and multiple scattering. The random spatial distributed physical composition of concrete also leads to the inhomogeneities of sound speed, which can distort and redirect the traveling wave. Previous studies have shown how an optimal focusing is achieved through inhomogeneous media, using the time-reversal invariance of wave equations. The TR focusing process acts as a spatial-temporal matching filter [38]. Its self-adaptive character can compensate for the distortion caused by the propagation through heterogeneous media.

The aggregate of concrete can be approximately equivalent to a scatterer, which results in information loss of the signals whose wavelengths are smaller than the classical diffraction limit (diameter-to-wavelength ratio of 0.5). Given the P-wave speed of 4000 m/s, when the governing frequency is set as 150 kHz, the calculated wavelength is 26.7 mm. Considering a maximum concrete aggregate size *d_max_* = 5 mm (diameter-to-wavelength ratio of 0.19 < 0.5), low scattering herein is expected. The numerical simulation also shows the feasibility of robust focusing through inhomogeneous media when the propagating wavefield generated by the crack is dominant over the noise.

### 2.3. Principle of the Time Reversal Method

**Figure 3:**
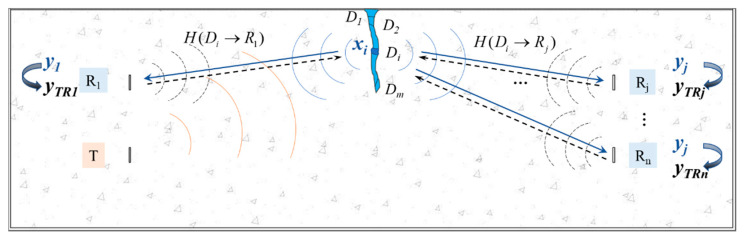
A sketch map of the wave time reversal process.

The principle of TRM in a concrete beam basically contains two parts: forward simulation and inverse simulation. The forward simulation aims to extract the damage-related signal to be focused, which is used as the input signal in the following reversal simulation. As shown in Figure 3, a transducer *T* and *n* receivers *R_*1*_, R_*2*_, ..., R_n_* are embedded in the test specimen, with a crack divided into *m* point-like areas *D_*1*_, D_*2*_, ..., D_m_*. The propagation wave is emitted by a transducer *T*, then the damage-related wave is extracted by differentiating the wave field of the damaged beam from the healthy one, which originates from the crack. The inverse simulation is a re-emission process. The damage-related signal is time reversed and imported into the numerical model to re-emit from receivers *R_*1*_, R_*2*_, ..., R_n_*. As a result, the damage-related signals are focused on each point-like area *D_i_* at different time steps. Then, the whole crack area is obtained by accumulating the focus results by time.

The input signal *x_i_*(*t*) is generated from damage point *D_i_*, then the received signal at *R_j_* in the time domain is:

(1) $$y_{j}\left(t\right)=x_{i}(t)⊗H(t,D_{i}\to R_{j})$$

where ⊗ is the convolution operator. *H* (*t,* *D_i_→R_j_*) is the transfer function from the damage point *D_i_* to the receiver *R_j_*.

If the signal is reversed and re-emitted in the time domain, the received signal at any point *P* in the control domain is:

(2) $$y_{P}\left(-t\right)=H(t,R_{j}\to P)⊗y_{j}(-t)=x_{i}(-t)(H(-t,D_{i}\to R_{j})⊗H(t,R_{j}\to P))$$

For the wave source where *P* = *D_i_*, according to the symmetry of the transfer function that *H* (*t, R_j_→D_i_*) = *H* (*t,* *D_i_→R_j_*), a constructive temporal coherence is obtained as follows:

(3) $$y_{Di}\left(-t\right)=x_{i}(-t)(H(-t,D_{i}\to R_{j})⊗H(t,D_{i}\to R_{j}))$$

Equations (2) and (3) can be expressed in the frequency domain:

(4) $$y^{*}{}_{P}\left(\omega \right)=x^{*}{}_{i}(\omega )H^{*}(\omega ,D_{i}\to R_{j})H(\omega ,R_{j}\to P)$$

(5) $$\begin{array}{ll} y^{*}{}_{Di}\left(\omega \right) & =x^{*}{}_{i}(\omega )H^{*}(\omega ,D_{i}\to R_{j})H(\omega ,D_{i}\to R_{j})\\ & =x^{*}{}_{i}(\omega ){\left|H(\omega ,D_{i}\to R_{j})\right|}^{2} \end{array}$$

where * denotes the complex conjugate of the function, and *ω* is the angular frequency. *y_P_* (*ω*), *y_Di_* (*ω*), and *x_i_* (*ω*) are the Fourier transforms of the signals *y_P_* (*t*), *y_Di_* (*t*), and *x_i_* (*t*), respectively.

Let the whole crack *D_i_* (*I* = 1, 2, ..., *m*) emit the damage signals and the receiver array *R_j_* (*j* = 1, 2, ..., *n*) re-emit the input signals. The output signal at a given damage area *D_k_* (*k* = 1, 2, ..., *m*) can be expressed as:

(6) $$\begin{array}{ll} y^{*}{}_{k}\left(\omega \right) & =\sum_{j=1}^{n}H(\omega ,R_{j}\to D_{k})y^{*}{}_{j}\\ & =\sum_{j=1}^{n}H(\omega ,R_{j}\to D_{k})\left[\sum_{i=1}^{m}x^{*}{}_{i}(\omega )H^{*}(\omega ,D_{i}\to R_{j})\right]\\ & =\sum_{j=1}^{n}\left[x^{*}{}_{i}(\omega ){\left|H(\omega ,D_{k}\to R_{j})\right|}^{2}+\sum_{\begin{array}{l} i=1\\ i\neq k \end{array}}^{m}x^{*}{}_{i}(\omega )H^{*}(\omega ,D_{i}\to R_{j})H(\omega ,R_{j}\to D_{k})\right] \end{array}$$

From the above formula, we can find that for all the damage signals diverging from source *D_k_*, their reversed waves can only converge in the original source *D_k_* among all the complex paths, while a poor spatial coherence is obtained in the other area due to the interference. This phenomenon can also be explained by the time-reversal invariance of the wave equations.

### 2.4. Wavelet Packet Decomposition and Damage Index

Wavelet packet analysis is an effective tool in signal processing, which can decompose a time-domain signal into different frequency components and evaluate the sensitivity of different frequency bands to damage. In this paper, the wavelet packet is introduced to calculate the energy of the signal, and the damage index is obtained to analyze the energy attenuation of cracks of different depths, and then to be compared with the imaging results.

The sensor signal *S* is decomposed into 2^n^ signal sets as {*X*_1_, *X*_2_,…, *X*_2_^n^}, where each signal can be presented as

*X_j_* = [*X_j_*_,1_, *X_j_*_,2_,…, *X_j_*_,*m*_] (7)

where *j* is the frequency range (*j* = 1, 2, …, 2*^n^*), *m* is the number of samples contained in the signal, and *n* is the level of wavelet packet decomposition, where *n* = 3 is herein assumed.

The energy of each signal is calculated as:

(8) $$X_{j}=\sum_{k=1}^{k=m}X_{j,k}$$

The energy of the received signal of the healthy beam *E_h_* and of the damage beam *E_d_* can be expressed as:

*E_h_* = [*X_h_*_,1_, *X_h_*_,2_, …, *X_h_*_,*2*_*^n^*] (9)

*E_d_* = [*X_d_*_,1_, *X_d_*_,2_, …, *X_d_*_,*2*_*^n^*] (10)

The energy-based damage index can be presented in the form of the root-mean-square deviation (RMSD) as:

(11) $$DI=\sqrt{\frac{\sum_{j=1}^{2^{n}}{\left(E_{d,j}-E_{h,j}\right)}^{2}}{\sum_{j=1}^{2^{n}}E_{h,j}{}^{2}}}$$

## 3. Numerical Modeling Based on the Finite Element Method

### 3.1. Property and Modeling Principle of Piezoelectric Patch

For linear elastic materials, the mechanical and electrical behavior is governed by the following piezoelectric constitutive equations in the matrix form:

(12) $$\begin{array}{l} \sigma =C^{E}:\varepsilon -e\cdot E\\ D=e^{T}:\varepsilon +\xi^{\varepsilon }\cdot E \end{array}$$

where σ and ε are the mechanical stress and strain tensor, ***D*** is the vector of electric displacement, ***C^E^*** is the elastic stiffness matrix, ***E*** is the electric field intensity vector, ***e*** is the piezoelectric stress factor matrix, and $\xi^{\varepsilon }$ is the dielectric constant matrix.

When the piezoelectric patch is exposed to the coupled stress-electric field, the equilibrium equation of the piezoelectric material is derived using the virtual displacement principle. In FEM, displacements and electric potentials are interpolated by nodal quantities, then the equilibrium equation is derived in the following matrix form:

(13) $$\left(\begin{array}{cc} K_{uu} & K_{u\varphi }\\ -K_{u\varphi } & K_{\varphi \varphi } \end{array}\right)\left(\begin{array}{l} U\\ \Phi \end{array}\right)=\left(\begin{array}{l} F_{u}\\ \rho_{\varphi } \end{array}\right)$$

where $K_{uu}$, $K_{\varphi \varphi }$, and $K_{uu}$ are the mechanical stiffness, dielectric constant, and piezoelectric coupling matrix, respectively. $F_{u}$ is the mechanical force and $\rho_{\varphi }$ is the electric charge volume density.

Due to the high sensitivity and low cost, the PZT-5A patch is selected as the core transducer of a CPIM, the dielectric property of which is defined by the permittivity values *D*_11_ of 424, *D*_22_ of 424, and *D*_33_ (in polar direction) of 450. The mechanical property is presented by the stiffness matrix as follows:

(14) $$\left(\begin{array}{cccccc} 12.6 & 8.41 & 7.95 & 0 & 0 & 0\\ 8.41 & 12.6 & 8.41 & 0 & 0 & 0\\ 7.95 & 8.41 & 12.6 & 0 & 0 & 0\\ 0 & 0 & 0 & 2.3 & 0 & 0\\ 0 & 0 & 0 & 0 & 2.3 & 0\\ 0 & 0 & 0 & 0 & 0 & 2.3 \end{array}\right)\times 10^{10}(N/m^{2})$$

The tie constraint surface contact is used to define the contact between the concrete and the piezoelectric patch. More information of the piezoelectric patch model is presented in [26].

### 3.2. Random Aggregates Model

To describe the heterogeneity of concrete components (such as the geometry, elastic properties, and spatial distribution), a random aggregate model is established by the secondary development of ABAQUS software based on Python. The random aggregate structure consists of two parts: the randomly distributed concrete aggregates and mortar filling the remaining area. The model is generated by the following steps:

Step 1 —estimate the particle size distribution. The random sampling principle of the Monte Carlo method is applied to obtain the particle size distribution in the simulation, following the grading curve with a maximum grain size of 5 mm.
Step 2 —determine the geometry of each aggregate. First, a circular particle size is generated with the size from step 1. Then, it is cut into a polygon, whose side length and inner angle are controlled by the irregularity parameter.
Step 3 —release the aggregates into the beam. Before placing the aggregates, the beam should be meshed into structured grids. The grid size is considered to be 1mm to achieve a good balance of image resolution and computing cost. The aggregates are then randomly mapped to the grid in the order from large ones to small ones. It is also necessary to check the potential problems, such as aggregate overlap, boundary intersection, and gaps between cement faces. If the above problems occur, the aggregates should be re-dropped.

**Figure 4:**
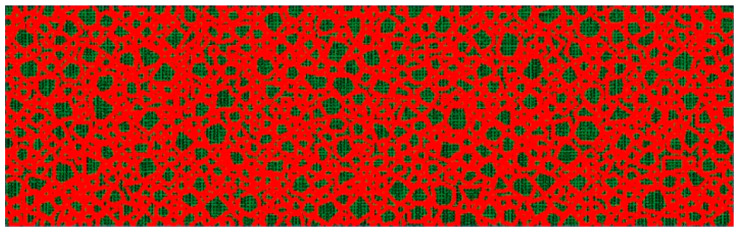
Random aggregates model.

An obtained random aggregates model is presented in Figure 4.

### 3.3. Modeling of Wave Propagation

The dynamic explicit algorithm adopts the time difference scheme of the dynamic equation. It is effective to solve non-linear dynamic problems, such as wave propagation modeling in an inhomogeneous medium, due to the fact that it does not need to iterate or to solve the tangent stiffness directly. The stability of the explicit model depends on the time increment Δ*t*, which should be small enough to satisfy the Neumann criterion. The quadrangle unit is herein used and the element size has a great influence on the calculation accuracy and computing cost. Based on the simulation theory, the number of finite elements *n* per wavelength should not be less than 10 [39]. Supposing that the wavelength is 26.7 mm, the structured element size is chosen as 1 mm (26.7 elements per wavelength). The forward mesh method is adopted, and the time increment is set as 0.2 μs. To reflect the change of the wave field caused by crack rather than meshes, the elements remain the same for the damaged and healthy beam, while the crack and PZTs are modeled by assigning corresponding materials to prescribed finite elements. The material parameters are shown in Table 1.

#### 3.3.1. Forward Simulation

**Figure 5:**
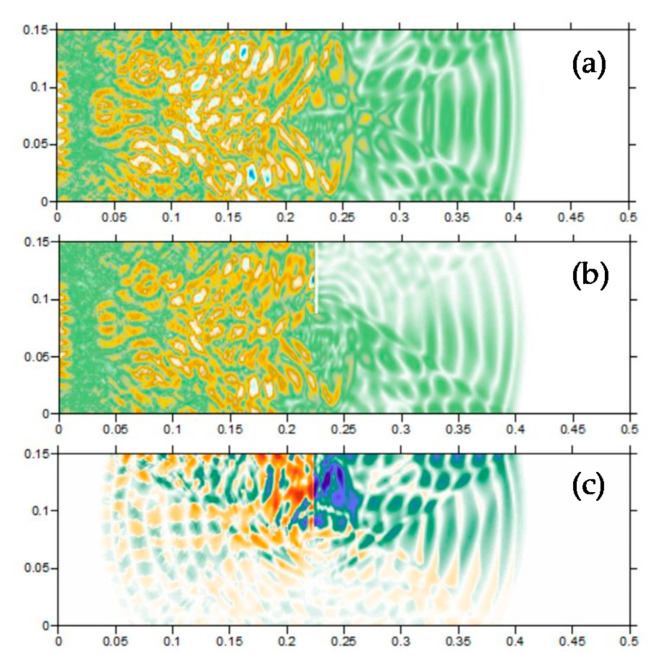
Wave field in homogeneous models: (**a**) healthy beam; (**b**) damaged beam; (**c**) The difference wave field of the healthy and damaged beam.

**Figure 6:**
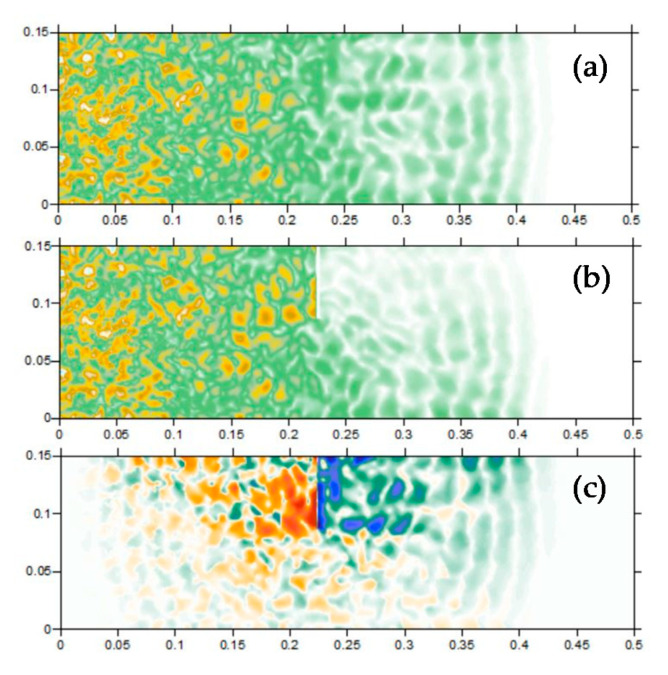
Wave field in the random aggregates models: (**a**) healthy beam; (**b**) damaged beam; (**c**) The difference wave field of the healthy and damaged beam.

A forward numerical model is established to simulate the fields and analyze the influence of the crack. The dimension of the concrete beam model is 0.5 × 0.15 × 0.15 m. An artificial crack is set at mid-span, locating from the top surface and extending to the central axis of the beam. A piezoelectric actuator is embedded on the left side of the beam along the central axis, which stimulates sine signal. Then, the horizontal displacement field is obtained under three states: (a) healthy, (b) damaged, and (c) the difference of the healthy and damaged beam, see Figure 5a–c. In order to analyze the influence of concrete inhomogeneity on the wave field (high-order scattering, inhomogeneities of sound speed, distortion etc.), a contrast heterogeneous wave propagating model was established, as shown in Figure 6a–c.

For the healthy state shown in Figure 5a, the wave fronts travel horizontally and undisturbed, and the corresponding displacements at nodes of CPIMA are used as the baseline. In Figure 5b, we can see the obvious attenuation of the propagating wave caused by the crack, as well as the slight “leakage” of the propagating wave caused by water filling in the crack as a media. The comprehensive influence of the crack on the wave field can be seen in Figure 5c, which is a snapshot of the difference of the displacement field of Figure 5a,b. By differencing, the damage signal is extracted as if it is generated by the crack, including three components: (1) the attenuated wave, which travels right; (2) the reflected wave, which travels left; and (3) the diffraction wave, which diffuses around from the crack tip. To capture all the signal components, the modules are suggested to be as more as possible and embedded along the boundary of the monitoring region to capture waves in all directions from the crack.

It can be seen from Figure 6a–c that the inhomogeneities of the media can distort and scatter the wave field. However, this distortion cannot redirect the wave fronts and the scattering effect is not strong enough to overwhelm the wave path from the wave source (crack) to the piezoelectric sensors. The wave reversibility to the crack is valid and hence TRM is adaptive for concrete media.

#### 3.3.2. Inverse Simulation and Imaging Conditions

The inversion model simulates the focusing process of the backward wave field. Crack 1 was introduced as an example to the random aggregates FE model, whose location is shown in Table 2. Three piezoelectric actuators of the array are selected to re-emit the damage signal after the time reverse process. Based on the elastic wave theory, the energy density can be separated by two parts: P-wave (rotation free part) and S-wave (isochore part). Hence, three imaging conditions can be developed using P-wave energy, S-wave energy, and total energy density. For the good orientation, the P-wave energy-based imaging conditions are chosen in the inverse simulation.

**Figure 7:**
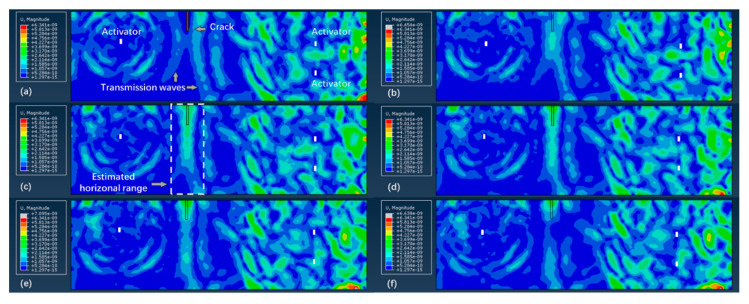
Focus result of the first wave fronts at different time instants: (**a**) 54.07 μs; (**b**) 56.16 μs; (**c**) 58.08 μs; (**d**) 59.04 μs; (**e**) 60.00 μs; (**f**) 61.13 μs.

Figure 7a–f are snapshots of the typical focusing moments of the first P-wave fronts from 54.07 to 61.125 μs, from which we can see the P-wave fronts interfere constructively at different damage areas in different time steps. Separation of the S-wave and P-wave is also observed due to the difference in the velocity and direction. In some snapshots, the focus areas do not match the crack accurately; however, the accuracy can be improved by accumulating the energy values of displacement during the focusing period.

To display the energy field, the energy density of the P-wave at the position ***x*** is calculated as [40]:

(15) $$E(x)=\sum_{t=0}^{t=T}(\lambda +2\mu ){\left[\nabla \cdot u(x,T-t)\right]}^{2}$$

and normalized energy density is obtained:

(16) $$\hat{E}(x)=\frac{E(x)-\bar{E}(x)}{\underset{x\in \Omega }{\max}E(x)-\bar{E}(x)}$$

where *u* (***x***, *T* − *t*) is the displacement at location ***x*** at time step *T* − *t*. *T* is the total focusing time period. Ω is the detection domain. $\bar{E}(x)$ is the average value of the energy E(x) in the monitoring region.

The different image conditions (energy calculation) have a great influence on the imaging results. In a previous study, there are two typical energy calculation methods based on the maximum energy value and the accumulated the energy by time [32,36]. The maximum energy is obtained by combining the maximum energy density of each node during the entire focusing period together, or recording the energy distribution when the overall maximum energy density appears. Considering the complex service environment and the background noise of underwater conditions, the accumulation energy method is applied to improve the robustness of the imaging method. For CPIMA with *n* piezoelectric modules, *n* tests are conducted for the detection of each crack. Each piezoelectric module is chosen as an actuator in turn from the first to the last, while the rest function as sensors. Hence, the average of *n* groups of energy density is used as the image condition ${\hat{E}}_{avg}(x)$.

## 4. Experimental Study

### 4.1. Experimental Setup

**Figure 8:**
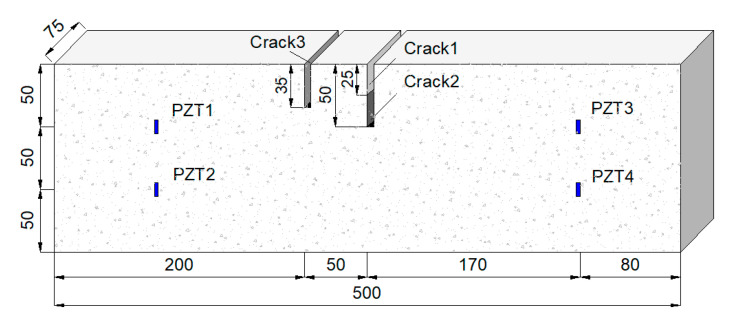
Layout of the cracks and sensor array.

A total of three concrete specimens were studied, with the strength of the cement of 32.5 MPa and a mixture ratio of water, cement, fine sand, and stones of 0.44:1:1.42:3.17. The diameter of coarse aggregates was 3 mm~5 mm, and the size of the specimens was 500 mm × 150 mm × 150 mm. Four piezoelectric modules (PZT1~4) were fixed in each concrete beam before pouring. All piezoelectric modules were located on the neutral surface of the specimen (see Figure 8), with a maximum horizontal distance of 340 mm and a vertical distance of 50 mm.

After the curing period of 28 days, each concrete specimen was first tested in the healthy state to get reference signals by recording the received signals, which were propagated from the piezoelectric actuator and captured by the remaining three piezoelectric sensors. Note that the actuator was chosen in turn from PZT1 to PZT4, hence there were four groups of reference signals for each specimen. Then, each of the three specimens was cut into an artificial crack of crack 1, crack 2, and crack 3, respectively. The crack setup is shown in Table 2 and Figure 8. The damaged signals could be obtained by testing the cracked concrete specimen in the same manner. During the test, the specimens were immersed in water.

**Figure 9:**
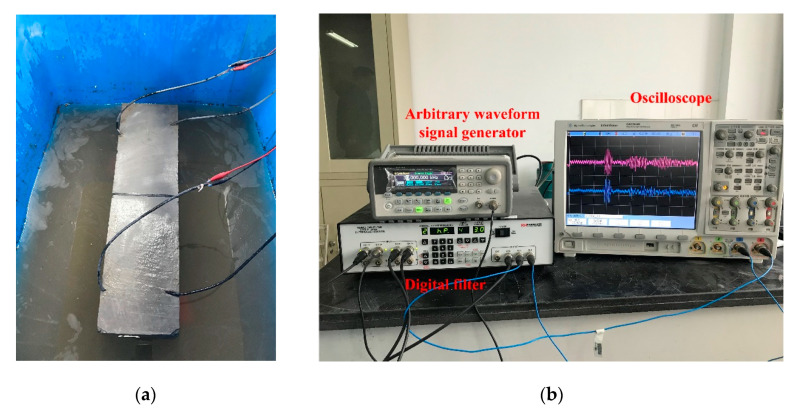
Experimental setup for underwater crack detection: (**a**) the specimen immersed in water; (**b**) experimental devices and the connections.

The Agilent 33522A arbitrary waveform signal generator was employed to send signal to the piezoelectric actuators. A digital filter was applied to filter the low-frequency AC noise. Signals were recorded by an Agilent DSO7034B oscilloscope with a bandwidth of 350 MHz and a sampling frequency of 3 MHz. The experimental setup is shown in Figure 9.

**Figure 10:**
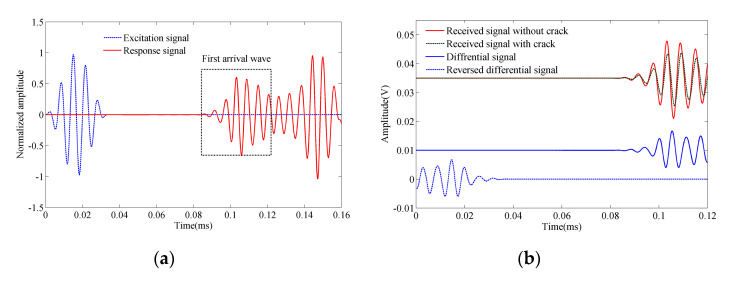
Extraction of the damage signal: (**a**) Excitation signal and response signal; (**b**) received signal of the beam with/without crack, their differential and reversed signal.

A 5-cycle 150 kHz Hanning-windowed tone burst, with a maximum amplitude of 10 V, was applied as the excitation signal. In order to reduce the influence of the reflected waves from the external boundary, the first arrival wave of the response signal was studied. The experimental data used were filtered by wavelet analyzer to extract signals of the dominant frequency of 150 kHz. The normalized excitation and response signal are shown in Figure 10a. The focus period was 0.24 ms, and the forward period and inverse period were both 0.12 ms, respectively. As shown in Figure 10b, the red line presents the received signal of the healthy beam, which was used as a reference. The green line is the received signal of the damaged beam. The blue line is the difference between the received signal with the crack and the reference signal, which presents the crack-related signal. The blue dash line is the time-reversed crack-related signal, which is used as the input signal in the inverse model.

### 4.2. Experimental Procedures

**Figure 11:**
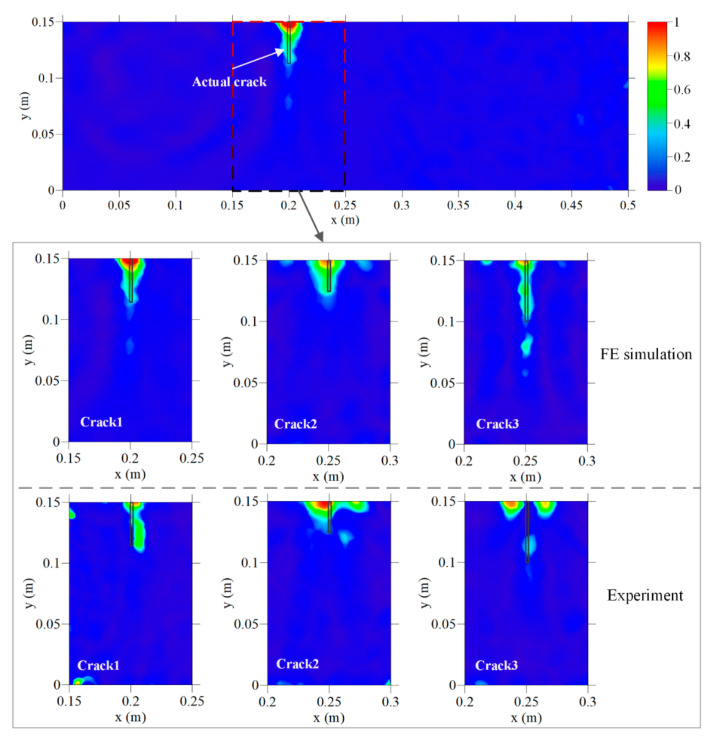
Imaging results of underwater crack by FE simulation and the experiment based on ${\hat{E}}_{avg}(x)$ at a dominating frequency of 150 kHz.

The experiment consists of three steps: (1) Forward process: Each of the three healthy beams were first placed underwater and tested to get reference signals. The depth of the water was about 0.15 m to precisely immerse the beam and avoid the effect of water pressure. After the test, the beam was carried out of the water to prevent water seepage in the concrete. Then, each beam was cut by a sewing machine into crack 1, crack 2, and crack 3, respectively. The cracked beams were immersed in water and tested again to get the damaged signals; thus, damage-related signals were extracted. The entire test process was finished within one week to avoid the effect of temperature change; (2) Inversion process: The first arrival wave of the time damage-related signals was reversed in the time domain and was imported as input signals in the inverse model. The obtained displacement and energy field were recorded; (3) imaging process: The energy value of each node was superimposed and converted to a true-color colorful cloud image after normalization to visualize the crack, see Figure 11.

**Figure 12:**
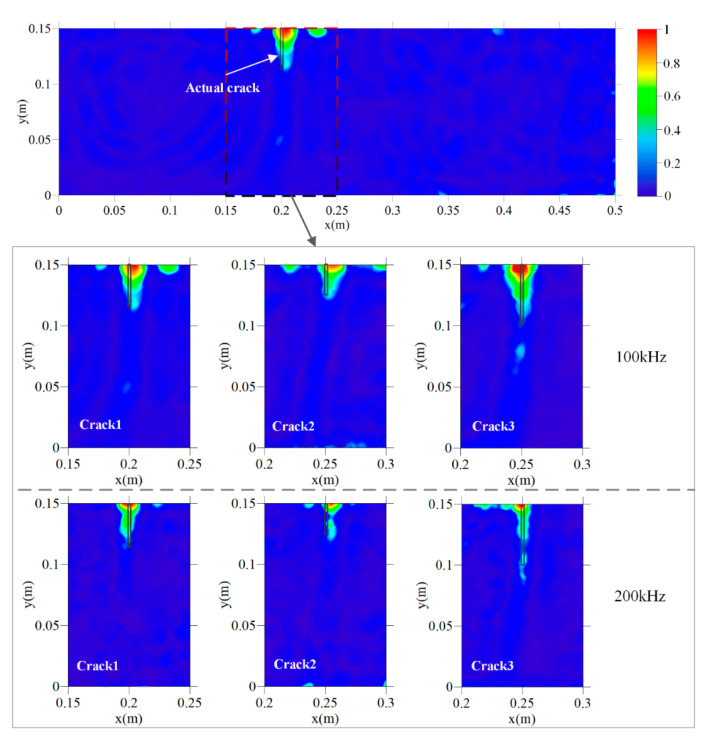
Imaging results of the underwater crack by FE simulation based on ${\hat{E}}_{avg}(x)$ at the dominating frequencies of 100 and 200 kHz.

In order to verify the effectiveness of the proposed method and its robustness to noise, the above process was simulated by FE models as a comparison, and the imaging results are shown in Figure 12.

### 4.3. Results and Discussion

The imaging results include colors from dark blue to red consistent with the average energy density ${\hat{E}}_{avg}(x)$ from low to high, see Figure 11. Due to the reflection of the beam surface, the crack opening is usually intensively focused, represented by the area where the maximum energy density appears. A threshold level of 30% of the normalized maximum energy density was set to extract the damage areas. Considering that the crack shape is rectangle, the quantitative identification results of the crack can be measured by the crack opening position and crack depth, see Table 3.

Figure 11 shows that underwater cracks, with different locations and depths, are imaged by the proposed TRM-based detection method, demonstrating that the extracted damage-related waves can carry the crack information and can be focused in the damage area through heterogeneous media (concrete). Additionally, the effect of the transmitted waves through water-filled-in cracks has been considered as a part of the attenuated wave component of the damage-related waves, hence the function of the crack as a wave source will not be influenced by the water. Except crack 3 in the experiment group, all the cracks in both the FE simulation group and experiment group are basically covered by the identified damage areas with shapes consistent with the direction of the actual cracks.

In the FE simulation group, note that the width of the identified damage area is wider near the crack opening with higher energy density, while the opposite phenomenon can be observed near the crack tip. This rule is more obvious for cracks with a shorter depth. For crack 2, crack 1, and crack 3, the width of the identified damage areas on the upper edge is 30, 25, and 18 mm, respectively. This is because for surface cracks with a shorter depth, a higher proportion of the crack-initiated waves is reflected by the surface of the concrete beam during propagation, making the surface a strong wave source comparable to the wave strength emitted from the crack. For crack 3, the identification of a deep surface crack is not seriously influenced by the reflected waves from the upper concrete surface; therefore, the energy density is equally distributed and the identified width is always about 18 mm along the vertical direction. However, an artifact appears about 20 mm below the crack tip. There are two possible reasons to explain this: The strong scattering of an exceptionally large concrete aggregate, or the information loss. From the random aggregates model (see Figure 4), no concrete aggregate with an exceptional large size is observed near the crack tip area, hence the possibility of strong scattering is excluded. Another reason is the information loss, which is caused by the layout and the number of piezoelectric modules employed. Due to the limited size and the shape of the beam specimen, all the four piezoelectric modules are embedded close to the middle line of the concrete beam, and their polar directions are parallel to the middle line of the beam. Therefore, only the information of waves propagating almost horizontally is available (see Figure 7). In addition, the useful information is limited by the number of sensor modules since the damage-related information is available at the sensors’ location only. In the authors’ opinion, this problem can be solved by placing the piezoelectric modules symmetrically on the boundary of the monitoring area and put them in multiple directions.

For the experimental imaging results, all three cracks are successfully detected, and the imaging results of crack 1 and crack 2 have good consistency with the actual ones. Crack 3 can also be identified from the image; however, the energy density of is unevenly distributed in the damage area due to the environmental effect, such as electromagnetic interference.

Table 3 shows that the crack opening position in the horizonal direction is accurately identified in both the FE simulation and experiment groups. Except crack 3 in the experiment group (11 mm), all the errors of the crack opening position are less than 4 mm. Note that the wavelength of the P-wave used herein is about 26.7 mm (150 kHz frequency), and the errors of the crack opening position are much smaller than the wavelength. This indicates the possibility that the resolution of the wave-based method can be increased by TRM focus. In both the FE simulation and experiment groups, the relative errors of crack depth identification are under 12%, except the crack 3 in the FE simulation group (37.8%). However, the error of crack 3 is caused by the artifact. If the effect of artifact is eliminated, the relative error and error would be 3.8% and 19 mm, respectively. To compare the errors of vertical depth with that of the horizontal position, the errors of the vertical depth are nearly one order larger than that of the horizontal position, showing that the lateral resolution is higher than the longitudinal resolution. Considering the wavelength (26.7 mm), the error of the crack depth is acceptable.

The energy attenuation-based detection method and RMSD damage index are very established in concrete crack detection. For underwater conditions, all the RMSD values are above 0.2, indicating the sensitivity to the existence of concrete cracks. Further, for crack 2 and crack 3 in the same position, the RMSD value is changed consistently with the increase of the crack depth. This shows that the energy-based RMSD damage index is an effective and reliable approach to the underwater concrete crack detection and can be a reference to TRM. However, the TRM imaging result is able to provide much more information about the crack than RMSD.

In FE simulation, the fineness of the mesh influences the resolution of the model, not only for shaping the aggregates and cementing surface but also for simulating the propagating wave fronts. However, since the higher element density will consume additional computing resources and the self-focusing character of TRM does not depend on the wave paths, extra fine mesh is unnecessary when Neumann criterion is satisfied. The element size of 1 mm is conformance to the resolution requirements of the presented frequency (100, 150, and 200 kHz) and the scale of the applied specimen (meter scale).

Based on the wave-based detection theory, the dominant frequency is one of the determining factors of detection resolution. For the frequency of 150 kHz, the wavelength (26.7 mm, centimeter scale) is one order larger than the actual crack width (1 mm, millimeter scale). As a result, the width of the identified crack area is usually lager than the actual one. In theory, higher frequency means higher resolution but will lead to more serious attenuation (material damping) and scattering in concrete. Concrete can be regarded as a low-pass filter, and for frequencies over 400 kHz, which is believed in [41] to be the cut-off frequency in concrete, the elastic wave is extremely difficult to propagate due to the high damping of concrete. In many concrete damage detection experiments, the commonly used maximum size of concrete aggregate is less than 20 mm. When dominating frequencies are above 200 kHz, the wavelengths (below 20 mm) are close to the maximum size of concrete aggregates and this will lead to serious scattering. Therefore, the influence of frequency on the resolution is complex, and the increase of frequency is not equivalent to the improvement of accuracy in practice. It is necessary to evaluate the performance of the imaging method at different frequencies.

To analyze the influence of frequency on the accuracy of the proposed method, crack 1, crack 2, and crack 3 were identified by FE simulation with the dominating frequencies of 100 and 200 kHz. The imaging results are shown in Figure 12 and the quantitative crack imaging results of the crack opening position and crack depth are shown in Table 4.

Figure 12 shows that all the cracks in both the 100-kHz group and 200-kHz group can be detected and basically covered by the identified damage areas. In the 100-kHz group, the widths of the identified damage areas are significantly larger than that of the 200-kHz group, showing that the imaging resolution is directly related to the dominating frequency. In crack 3 of both groups, the artifacts are also observed near the crack tips.

Table 4 shows that the crack opening position is accurately identified in both groups with errors (below 6 mm) substantially less than the wavelengths (20–40 mm). To compare the accuracy of the two groups, it can be observed that the accuracy of both groups is similar with respect to the horizontal opening position and vertical depth. Further, by comparing the quantitative imaging results in Table 3 and in the FE simulation group in Table 4, it can be concluded that similar accuracy is achieved at frequencies of 100, 150, and 200 kHz. This shows that the robustness to the frequency is in the presented frequency range, and an appropriate excitation frequency can be selected based on the specific working conditions, such as the scale of the specimen, maximum size of the concrete aggregate, and resonant frequency of PZT transducers.

## 5. Conclusions

In this study, a CPIMA monitoring system and TRM-based imaging technique were applied to detect and image underwater cracks of concrete structures. To overcome the challenge of water filled in the crack and to improve the application of TRM, the concept of damage-related waves was proposed. A heterogeneous FE numerical model, including the piezoelectric constitutive model and the wave propagation model characterized by displacement, was established. This model was used to simulate both the forward process and inverse process of TR focusing. The forward process depicts the wave propagation through healthy and damaged underwater concrete beams, showing the effectiveness of the proposed damage-related wave based TRM focus in underwater conditions and its robustness to heterogeneous material (concrete).

The inverse process simulates the backpropagating wave field, then the focusing results of different cracks were obtained. Three underwater concrete cracks, with different locations and depths, were imaged by the proposed TRM-based detection method by both FE simulation and experiment. The FE simulation results show the feasibility of the proposed method in heterogeneous media to detect underwater cracks. The reflection of the concrete surface near the crack opening has some influence on the shape of the identified areas but has little influence on the accuracy of the identification of crack opening. The influence of the dominant frequency on the resolution is complex, and for the presented different frequencies, the accuracy of the imaging results is similar. The experimental imaging results show that the underwater surface cracks in concrete beams are imaged by limited piezoelectric modules with an acceptable error, indicating the application potential in underwater crack detection of concrete structures. Various effects (water pressure, temperature, and seepage etc.) on the performance of piezoelectric modules and received signals need to be further studied before being adapted to in situ monitoring for underwater concrete structures.

## Figures and Tables

**Table 1:** Material parameters.

	Mortar	Aggregate	Piezoelectric Patch
Young’s modulus (GPa)	30	80	75
Poisson’s ratio	0.2	0.2	0.32
Density (kg/m^3^)	2400	2800	7500

**Table 2:** Crack setup.

	Crack 1	Crack 2	Crack 3
Crack length (mm)	25	50	35
Distance to the left side (mm)	250	250	200

**Table 3:** Quantitative identification results of the crack.

	Crack Opening Position		Crack Size			
FE Modeling	Distance to the Left Side (mm)	Error (mm)	Vertical Depth (mm)	Error (mm)	Relative Error (%)	RMSD
Crack 1	201	1	381	31	8.9	0.3472
Crack 2	248	−2	272	22	8.7	0.2398
Crack 3	249	−1	689	189	37.8	0.5288
**Experiment**						
Crack 1	203	3	389	39	11.2	0.2961
Crack 2	246	−4	257	7	3.1	0.2168
Crack 3	239	−11	447	53	10.4	0.3834

**Table 4:** Quantitative crack imaging results at the dominating frequency 100 and 200 kHz.

		Crack Opening Position		Crack Size		
100 kHz	Distance to the Left Side (mm)		Error (mm)	Vertical Depth (mm)	Error (mm)	Relative Error (%)
Crack 1	204		4	371	21	6
Crack 2	256		6	284	34	13.6
Crack 3	249		−1	693	193	38.6
**200 kHz**						
Crack 1	200		0	366	16	4.6
Crack 2	255		5	297	47	18.8
Crack 3	249		−1	621	121	24.2
